# Upregulated GRB7 promotes proliferation and tumorigenesis of Bladder Cancer via Phospho-AKT Pathway

**DOI:** 10.7150/ijbs.49410

**Published:** 2020-10-23

**Authors:** Yingchun Zheng, Yuanyuan Pei, Le Yang, Zhi Zeng, Jie Wang, Guie Xie, Lan Wang, Jie Yuan

**Affiliations:** 1 Department of Pathogen Biology and Immunology, School of Life Sciences and Biopharmaceutics, Guangdong Pharmaceutical University, Guangzhou 510006, China.; 2 Shenzhen Long-gang Maternal and Child Health Hospital Centralab, Shenzhen 518172, China.; 3 Department of Basic Medicine, Nanyang Medical College, Nanyang, Henan 473061, China.; 4 Department of Physiology, School of Life Sciences and Biopharmaceutics, Guangdong Pharmaceutical University, Guangzhou 510006, China.; 5 Guangdong Provincial Key Laboratory of Pharmaceutical Bioactive Substances, Guangdong Pharmaceutical University, Guangzhou 510006, China.; 6 KingMed School of Laboratory Medicine, Guangzhou Medical University, Guangzhou, Guangdong 510182, China.; 7 Zhongshan School of Medicine, Sun Yat-sen University, Guangzhou 510080, China.

**Keywords:** GRB7, proliferation, bladder cancer, AKT, G1/S

## Abstract

Growth factor receptor-bound protein 7 (GRB7) has been found closely related to the occurrence and development of various tumors, but its function in bladder cancer has not yet been elucidated. The study is aiming at investigating the expression and function of GRB7 in bladder cancer. The Cancer Genome Atlas (TCGA) database was selected to analyze mRNA levels of GRB7 in bladder cancer. RT-qPCR and Western blot were conducted to detect the expression of GRB7 in normal bladder epithelial cells, seven bladder cancer cell lines and eight pairs of malignant/nonmalignant bladder tissues. The role of GRB7 in tumor proliferation and tumorigenesis was explored by establishing stable cells, *in vitro* cell experiments and *in vivo* xenograft models. The molecular regulation mechanism of GRB7 in bladder cancer was investigated by treatment with AKT inhibitor. GRB7* mRNA* was upregulated in bladder cancer samples compared with that in normal tissue samples. Overexpressing GRB7 significantly promoted the proliferation and tumorigenesis of bladder cancer. However, silencing GRB7 played the retarding part. GRB7 promoted G1/S transition by activating the AKT pathway. Our results indicate that GRB7 plays an important role in promoting proliferation and tumorigenesis of bladder cancer.

## Introduction

Bladder cancer is a malignant tumor that occurs in the bladder mucosa and is the most common malignant tumor of the urinary system 1. Clinical examination methods for bladder cancer include routine urine examination, exfoliative cytology, urinary tumor marker examination, abdominal and pelvic B-ultrasound examination 2. According to the preliminary examination results, cystoscopy, intravenous urography, CT or MRI can be used for definitive diagnosis 3. Tumor marker detection, as a non-invasive test, can be applied to the diagnosis of tumors and can be used for the monitoring of recurrence and prognosis 4. Clinically, the analysis of specific genes in patients' urine exfoliated cells is beneficial for early diagnosis 5. The development of modern high-throughput technology has led to the discovery of more and more molecular markers of bladder cancer. For example, detection of bladder tumor antigen (BTA), nuclear matrix protein (BLCA-4), and telomerase significantly increased the sensitivity and specificity of bladder cancer diagnosis 6-8. Although carcinoembryonic antigen (CEA) and carbohydrate chain antigen 125 (CA-125) can also be used as indicators for the diagnosis of bladder cancer, the sensitivity and specificity of bladder cancer detection are currently not ideal 9, 10. Only by finding more biomarkers and therapeutic targets can we effectively improve the early diagnosis and prognosis of patients with bladder.

Growth factor receptor-binding protein 7 (GRB7) is a member of the GRB7 signal transduction protein family including GRB7, GRB10 and GRB14 11. The domain of GRB7 is extremely conserved and consists of an N-terminal proline-rich domain, a RA domain, a central pH domain, a BPS motif and a C-terminal SH2 (src homology 2) domain 12. The multi-domain structure of GRB7 determines that it must be able to participate in the transduction of multiple signal paths. It is known that GRB7 is involved in important cell growth regulators such as the Erythroblastic Leukemia Viral Oncogene Homolog (ErbB) receptor family, platelet-derived growth factor receptor, insulin receptor and RAS GTPase, suggesting that GRB7 plays an important role in cell survival and growth 13, 14. Numerous studies have also reported the important role of GRB7 in the malignant progression of tumors. Proliferation and migration of gastric cancer and oral squamous cell carcinoma are significantly reduced when the GRB7 gene is knocked out 15, 16. In invasive breast cancer, the GRB7 protein binds to invasive phenotypic markers such as overexpressing HER2 and highly amplified HER2 17. In cervical cancer, high expression of GRB7 promotes distant invasion of cervical cancer and inhibits apoptosis 18. However, the role of GRB7 in the malignant processes of bladder cancer is unknown.

In the present study, we find that GRB7 mRNA and protein expression are upregulated in bladder cancer. Overexpressing GRB7 promotes, while silencing GRB7 inhibits the G1/S transition in the cell cycle as well as tumorigenesis through phospho-AKT pathway. These results suggest that GRB7 may be a diagnostic marker and a valuable therapeutic target for bladder cancer.

## Materials and Methods

### Microarray data processing and Statistical analysis

The RNA detection values of 408 BC tissues and 19 adjacent normal bladder tissues were downloaded from the Cancer Genome Atlas (TCGA). Excel and MeV 4.9 software were used to sort out these data and Gene Set Enrichment Analysis (GSEA; GSEA 2.2.1, http://www.broadinstitute.org/gsea) was adopted to analyze them. Besides, chi-square test was used to judge whether there were differences in data between groups. In this study, a double tailed *p* value less than 0.05 was considered statistically significant.

### Cell lines and tissues

Seven bladder cancer cell Lines (HT-1197, UMUC3, 5637, RT4, J82, HT-1376, T24) were cultured in Dulbecco's Modified Eagle's Medium (DMEM; Gibco, Rockville, MD, USA), supplemented with 10% fetal bovine serum (HyClone, Logan, UT, USA). Primary normal bladder epithelial cells (Normal) and eight paired BC tissues (ANT: adjacent normal tissues; T: tumor) were kindly collected and presented by Dr. Yang for this study. As previously described 19, epithelial growth factor, bovine pituitary extract, and antibiotics were added to the keratinocyte serum-free medium (Invitrogen Life Technologies, Carlsbad, CA, USA) with the purpose of helping the adherent culture of these cells.

### Real-time Quantitative PCR (RT-qPCR) and Western blot analysis

Real-time Quantitative PCR (RT-qPCR) and Western blot analysis were performed following standard methods as mentioned 19. RNA extraction was conducted according to standard methods in the manufacturer's instructions, and the RT-qPCR was performed with the help of the Biosystems 7500 Sequence Detection system. The following primers were used: GRB7 (forward, 5'-GGTTTGGAGGACCACGAGTC-3'; reverse, 5'-CGGAAGACGAAGCGGCTATC-3'); and *GAPDH* (forward, 5'-ACCACAGTCCATGCCATCAC-3'; reverse, 5'-TCCACCACCCTGTTGCTGTA-3'). Considering about the variability in expression level, all the expression data were normalized to the geometric mean of the housekeeping gene *GAPDH*. The main antibodies adopted in the present study were as followed: anti-GRB7 (1:500, Santa Cruz Biotechnology, USA), anti-p-AKT^Ser473^ (1:800, Cell Signaling Technology, MA, USA), anti-p-AKT^Thr308^ (1:800, Cell Signaling Technol, MA, USA), anti-AKT (1:800, Cell Signaling Technol, MA, USA), anti-cyclin D1 (1:1000, BD Transduction Laboratories, USA), anti-CDK6 (1:1000, Sigma-Aldrich, St. Louis, MO, USA), anti-CDK4 (1:1000, Sigma-Aldrich, St. Louis, MO, USA), anti-p21^Cip1^ (1:1000, Sigma-Aldrich, St. Louis, MO, USA), anti-p27^Kip1^ (1:1000, Sigma-Aldrich, St. Louis, MO, USA), anti-pRB (1:1000, Sigma-Aldrich, St. Louis, MO, USA), anti-RB(1:1000, Sigma-Aldrich,St. Louis, MO, USA), anti-p-GSK3β (1:800, Cell Signaling Technol, MA, USA), anti-p-GSK3β (1:800, Cell Signaling Technol, MA, USA). Anti-β-actin monoclonal antibody (1:1000, Abcam, Cambridge, UK) was taken as a loading control.

### Vectors and retroviral infection

The full-length human GRB7 was subcloned into the pMSCV-retro-puro vector (Clontech, Palo Alto, CA). The forward PCR primer was 5'-AGATCTATGGAGCTGGATCTGTCTCCAC-3' and reverse PCR primer was 5'-GAATTCTCATCAGAGGGCCACCCGC-3'. Besides, under the help of RNA interference (RNAi) method, the GRB7 knock down bladder cancer cell lines was also established. To endogenously downregulate GRB7, two hairpin GRB7 siRNA oligonucleotides (sense, 5'-GATCCCGCGAGTCCAACGTGTACGTGTTCAAGAGACACGTACACGTTGGACTCGTTTTTTGGAAA-3'; antisense, 5'-AGCTTTTCCAAAAAACGAGTCCAACGTGTACGTGTCTCTTGAACACGTACACGTTGGACTCGCGG-3') were inserted into the subcloned *pSUPER.retro.puro* plasmid (Oligoengine, Seattle, WA). As described before, retroviral production and infection were performed 19.

### In vitro cell proliferation assay

In cell counting part, the established cells were seeded in 96-well plates with a density of 2×10^3^ cells/well. The cell proliferative activity was tested by 3-(4,5-Dimethyl-2-thia-zolyl)-2,5-diphenyl-2-H-tetrazolium bromide (MTT; Santa Cruz Biotechnology, Inc., Dallas, TX, USA) assay. After cultured for 24 h, 48 h, 72 h, 96 h, 120 h or 144 h, each well was added with 10 µl of MTT solution (5 mg/ml). After incubation with the MTT reagent at 37 °C for 4 h, cells were added with 150 µl dimethyl sulfoxide (DMSO) and then the absorbance was measured at 490 nm on a microplate reader (BioTek Instruments, Inc., Winooski, VT, USA). All tests have been repeated at least three times.

As for colony formation assays, 1000 cells per well were plated in six-well plates in triplicates and cultured for ten days before staining viable colonies by nitro blue tetrazolium (Sigma). All experiments were conducted in triplicate for each cell line.

### Anchorage-independent growth assay

500 cells were lightly blown and suspended evenly in 1 mL complete DMEM supplementary with 0.7% agarose. Then the suspension was all lightly plated into 6-well plates container which have been pre-covered with 1.5% agarose. Each group had 3 parallel samples. The cells were then incubated with 5% CO_2_ at 37 °C, replacing the medium every 2 days. 12 days later, the cells were counted by the help of an inverted microscope, and during this observation, any opaque spot bigger than 0.1 mm was regarded as one colony. Images were photographed and stored for later analysis.

### Flow cytometry analysis

5×10^5^ cells/well were seeded in 100-mm dishes and normal cultured for 48 hours. Then, cells were harvested and fixed with ice-cold 70% ethanol before being kept overnight at 4 °C. Next, they were washed with PBS, collected, and centrifuged at 1500 rpm for five minutes, and after that the cells were incubated by the use of bovine pancreatic RNase (20 μg/ml, Sigma) at 37 °C for 30 min and stained with propidium iodide (20 μg/ml, Sigma). Cells were further subjected to cell cycle analysis by FACSCanto II flow cytometer (BD Biosciences, San Jose, CA, USA), and the data were analyzed with the help of the FLOWJO software (Tree Star, Inc., Ashland, OR). All experiments were conducted repeatedly three times.

### Bromodeoxyuridine (BrdU) labeling and immunofluorescence assay

Cells in the logarithmic growth stage were seeded at the initial density of 2000 cells per well in 24-well plates with coverslips (Fisher, Pittsburgh, PA) placed inside the wells, and then the cells were allowed to attach for 72 h. BrdU (working concentration was 10 µM) was then added into each well and allowed to be incubated for 1h. Then the cells were washed twice in PBS, fixed in 100% methanol (chilled at -20 °C) for five min, permeabilized with 0.2% TritonX-100 for ten min followed with the addition of immunostained with an anti-BrdU antibody (Upstate, Temecula, CA, USA). A laser scanning microscope (Axioskop 2 plus, Carl Zeiss Co. Ltd., Jena, Germany) was taken for counting and taking typical pictures.

### Murine Xenograft Model

The *in vivo* experiments were performed as described before 19. All institutional and national laboratory animal care and use guidelines were followed. Non-obese diabetic/severe combined immunodeficiency (NOD/SCID) mice (4-5 weeks old, 18-20 g) were purchased from the Guangdong Medical Laboratory Animal Center (Guangzhou, Guangdong, China). Cells (5,000,000) in 0.25 ml PBS were injected into the s.c. in the flank. Each experiment was performed repeatedly at least twice. The tumor volumes were calculated according to the following formula: V=A×B2/2 (cm^3^), where A was the largest diameter (cm) and B is the smallest diameter (cm). On the day of 24, the animals were euthanized, and the tumors were excised and weighed.

## Results

### GRB7 is upregulated in bladder cancer

**Figure 1:**
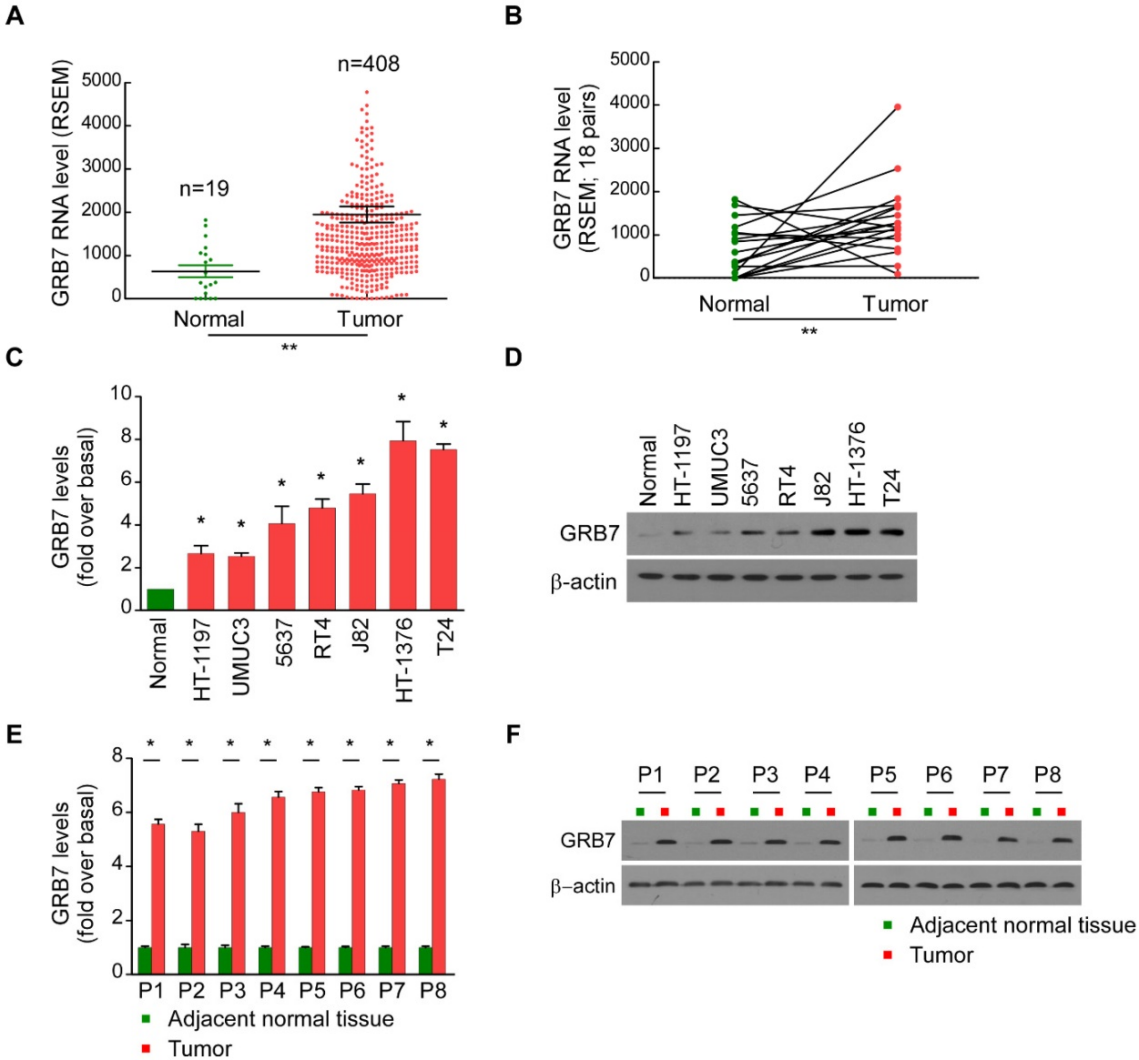
** GRB7 is upregulated in bladder cancer. A.** The mRNA level of *TEF* was frequently upregulated in bladder cancer tissues (Tumor) compared to that in normal bladder epithelial samples (Normal) in the TCGA database. Values are expressed as the mean ± SEM. **P < 0.001. **B.** The RNA levels of TEF were markedly decreased in 18 paired bladder cancer tissues (Tumor) compared to those in adjacent normal tissues (Normal) from 18 patients in the TCGA database. **P < 0.001. **C.** RT-qPCR analysis showed that the mRNA level of GRB7 was indeed upregulated in all of the seven cultured bladder cell lines compared to that in normal epithelial cells (Normal). **D.** GRB7 protein was also higher in the seven cultured bladder cell lines compared to that in normal epithelial cells.** E.** RT-qPCR analysis showed that the mRNA level of GRB7 were upregulated in malignant bladder cancer tissues (Tumor) compared to that in adjacent normal tissues. *P < 0.05. **F.** Western blot analysis revealed that the protein level of GRB7 were upregulated in malignant bladder cancer tissues compared to that in adjacent normal tissues.

The mRNA expression of GRB7 in 427 bladder cancer tissues in the TCGA database was analyzed. A point on the graph of Figure 1A represents the expression level of a sample. The GRB7 mRNA was upregulated in bladder cancer samples (Tumor) compared to that in normal tissue samples (19 cases, Normal) (*P* < 0.001). To exclude individual differences between patients, we further classified the 427 samples and obtained 18 pairs of bladder cancer tissues and adjacent normal tissues from the same patient. Among the 18 pairs of specimens, GRB7 mRNA was significantly higher in bladder tumors than in adjacent normal tissues (Figure 1B; *P* < 0.001).

For the experimental verification, seven bladder cell lines and five pairs of clinical bladder specimens were taken further detection. Compared with normal bladder epithelial cells, the mRNA levels of GRB7 in 7 cultured bladder cell lines were significantly increased (Figure 1C). The GRB7 protein was also higher compared to that in normal epithelial cells (Normal, Figure 1D). Meanwhile, RT-qPCR analysis and Western blot analysis revealed that the mRNA level and protein level of GRB7 were both upregulated in malignant bladder cancer tissues compared to that in adjacent normal tissues (Figure 1E and 1F). Taken together, GRB7 is upregulated in human bladder cancer indicated by these results.

### GRB7 regulates the proliferation of bladder cancer cells

**Figure 2:**
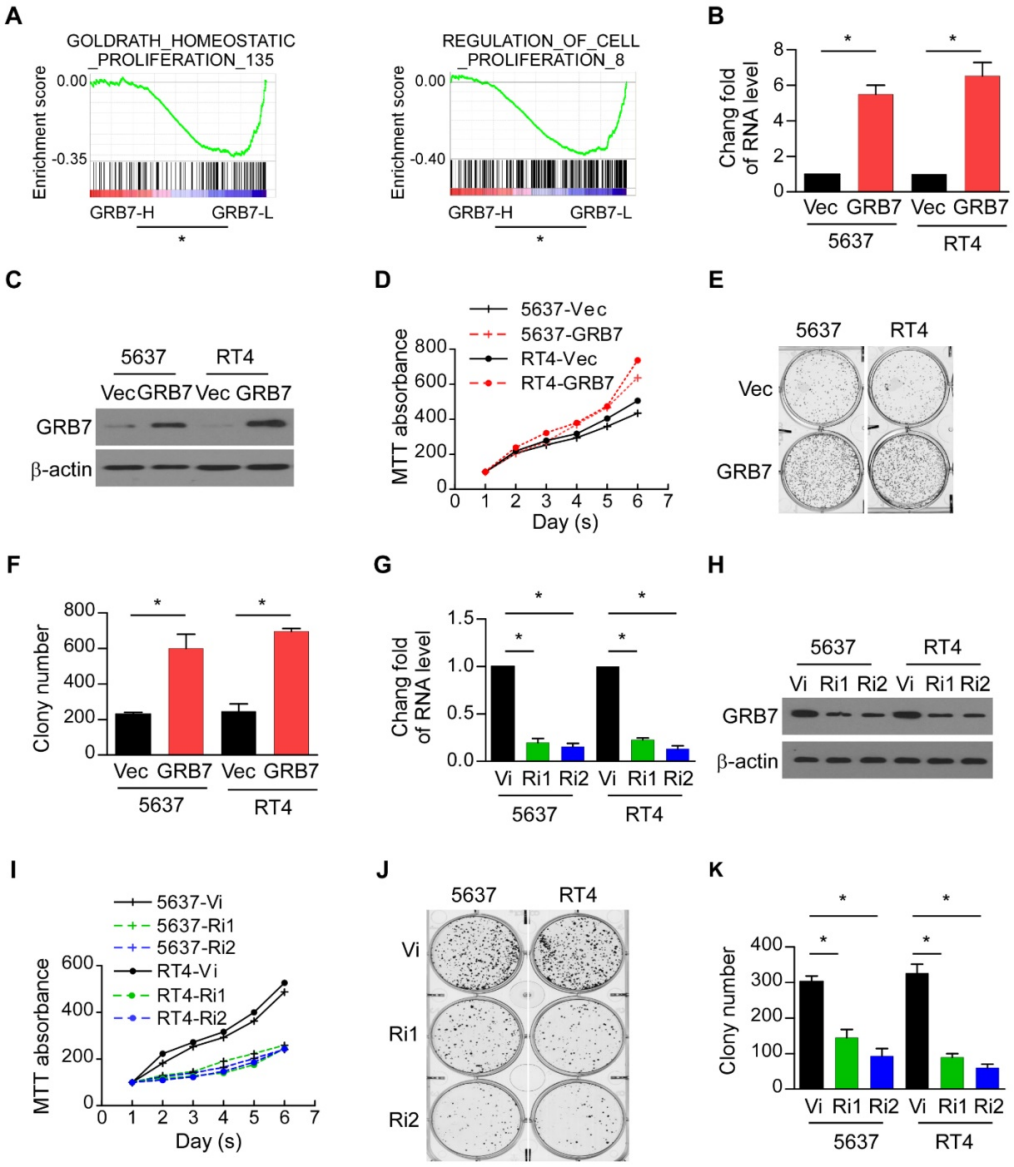
** GRB7 regulates the proliferation of bladder cancer cells. A.** GSEA plots demonstrated that the expression of GRB7 in bladder cancer is positively correlated with cell proliferation. *P < 0.05. **B.** RT-qPCR analysis showed that 5637 and RT4 cells were successfully and constantly transfected with GRB7 plasmids to overexpress the expression of GRB7. **C.** Western blot analysis showed that 5637 and RT4 cells were successfully and constantly transfected with GRB7 plasmids to overexpress the expression of GRB7. **D.** MTT assays showed that overexpression of exogenous GRB7 significantly increased the growth rate of 5637 and RT4 cells. **E.** A representative image from the colony formation assay showed that overexpression of exogenous GRB7 significantly increased the mean colony number in the colony formation assay.** F.** The mean count of the colony number in the colony formation assay. **G.** RT-qPCR analysis showed that 5637 and RT4 cells were successfully and constantly transfected with GRB7 plasmids to overexpress the expression of GRB7. **H.** Western blot analysis showed that the endogenous GRB7 were successfully silenced.** I.** MTT assays showed that silencing endogenous GRB7 significantly reduced the growth rate of 5637 and RT4 cells. **J.** A representative image from the colony formation assay showed that silencing endogenous GRB7 significantly reduced the mean colony number in the colony formation assay.** K.** The mean count of the colony number in the colony formation assay. *P < 0.05.

GSEA was used to assess the contribution of GRB7 to the malignant progression phenotype of the tumor. The mean value of GRB7 mRNA was used as the dividing line, and 408 cases of bladder cancer were divided into GRB7 high expression group (GRB7-H) and GRB7 low expression group (GRB7-L). Shown as Figure 2A, the expression of GRB7 in bladder cancer is positively correlated with cell proliferation (*P* < 0.05). For experimental verification, 5637 and RT4 cells were stably transfected with the GRB7 plasmid (GRB7 vs. Vec; Figure 2B and 2C). The MTT assay showed that overexpression of exogenous GRB7 accelerated the growth rate of 5637 and RT4 cells (Figure 2D). Cloning formation experiments showed that overexpression of exogenous GRB7 significantly increased the number of cell clones. (Figure 2E and 2F). In order to explore the role of endogenous GRB7 in cell proliferation, endogenous GRB7 was stably silenced in 5637 and RT4 cells (Ri1&Ri2 *vs.* Vi; Figure 2G and 2H). Silencing endogenous GRB7 not only slows the growth rate of cells but also reduces the average number of colonies (Figure 2I-K). These *in vitro* results indicate that GRB7 may play a role in promoting the proliferation of bladder cancer cells.

### GRB7 promotes the tumorigenesis of bladder cancer

**Figure 3:**
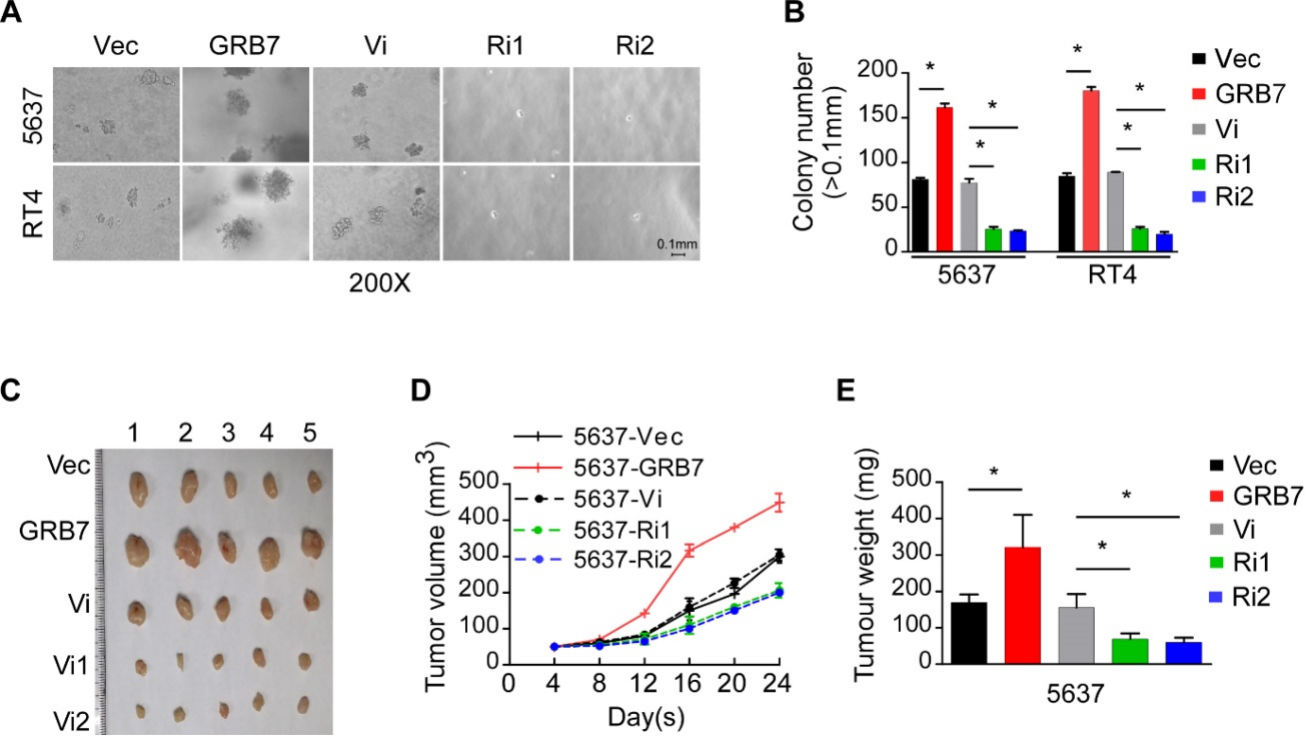
** GRB7 promotes the tumorigenesis of bladder cancer. A.** Representative images from the anchorage-independent growth assay show that the colony number was significantly increased in exogenous GRB7-overexpressed cells but decreased in endogenous GRB7-silenced cells. **B.** The mean count of the colony number in the anchorage-independent growth assay. **C.** Images of excised tumors from NOD/SCID mice which injection with 5637-Vector, 5637-GRB7, 5637-RNAi vector, 5638-RNAi1 or 5637-RNAi2 cells. **D.** Tumor volumes were measured every 4 days. Data represent the mean ± SD of three independent measures. **E.** Average weight of excised tumors from three different weighing instruments. *P < 0.05.

Anchorage independent growth assays showed that the number of clones increased significantly when GRB7 was overexpressed and significantly decreased when GRB7 was silenced (Figure 3A and 3B). The xenograft model established in NOD/SCID mice also validated the effect of GRB7 observed in *in vitro* experiments. By observing the growth rate of the transplanted tumor and weighing the final transplanted tumor, it was found that the tumorigenic ability of 5637 cells was significantly enhanced when GRB7 was highly expressed, but was greatly attenuated when GRB7 was silenced (Figure 3C-E). Taken together, the results indicate that GRB7 plays a positive role in the tumorigenesis of bladder cancer cells.

### GRB7 promotes the cell cycle G1/S transition in bladder cancer cells

**Figure 4:**
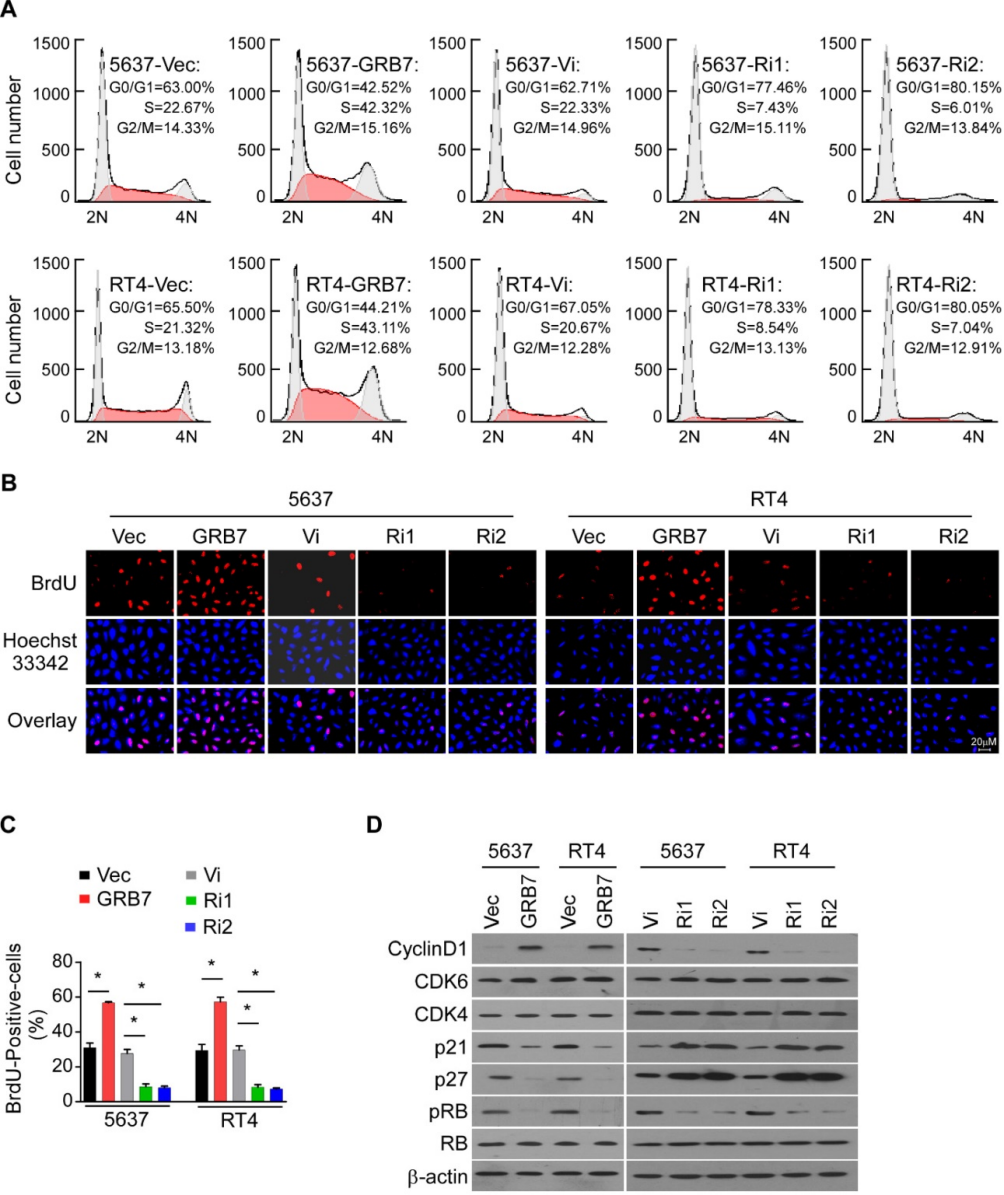
** GRB7 promotes the cell cycle G1/S transition in bladder cancer cells. A.** Flow cytometric analysis of stable cell lines constructed from 5637 and RT4 cells demonstrated that the percentage of S phase cells negatively correlated with the expression of TEF. **B.** Representative micrographs of BrdU showed that the percentage of BrdU-incorporated cells were higher in GRB7-overexpressed cells but lower in GRB7-silenced cells. **C.** the quantification of BrdU incorporation in stable cell lines. Three independent experiments were conducted. **D.** Western blot analysis of G1/S transition-associated genes showed that the protein expression levels of the cell cycle promoter is positively expressed with GRB7 expression, while the protein expression levels of the cell cycle inhibitor is negatively expressed with GRB7 expression. *P < 0.05.

Since cell growth is closely related to the cell cycle, flow cytometry was used to detect the effect of GRB7 expression level on the proportion of cells in each cycle. Overexpression of GRB7 significantly reduced the proportion of cells in G0/G1 phase and increased the proportion of cells in S phase; while silencing GRB7 significantly increased the proportion of cells in G0/G1 phase but decreased the proportion of cells in S phase (Figure 4A). In addition, representative images and statistics show that the BrdU-incorporated cell ratio is elevated in cells overexpressing GRB7 and decreased in cells silencing GRB7 (Figure 4B and 4C). Several key factors regulating cell cycle were examined here to observe the effect of GRB7 on cell cycle transition. As shown in Figure 4D, the proteins of the cell cycle promoters Cyclin D1, CDK4 and CDK6 were up-regulated in GRB7-expressing cells and down-regulated in GRB7-silencing cells. In contrast, the cell cycle inhibitors p21^Cip1^ and p27^Kip1^ proteins were decreased in GRB7 overexpressing cells and elevated in GRB7 silencing cells. These results clearly indicate that GRB7 promotes the cell cycle G1/S transition in bladder cancer cells.

### Phospho-AKT pathway is related to GRB7 expression

**Figure 5:**
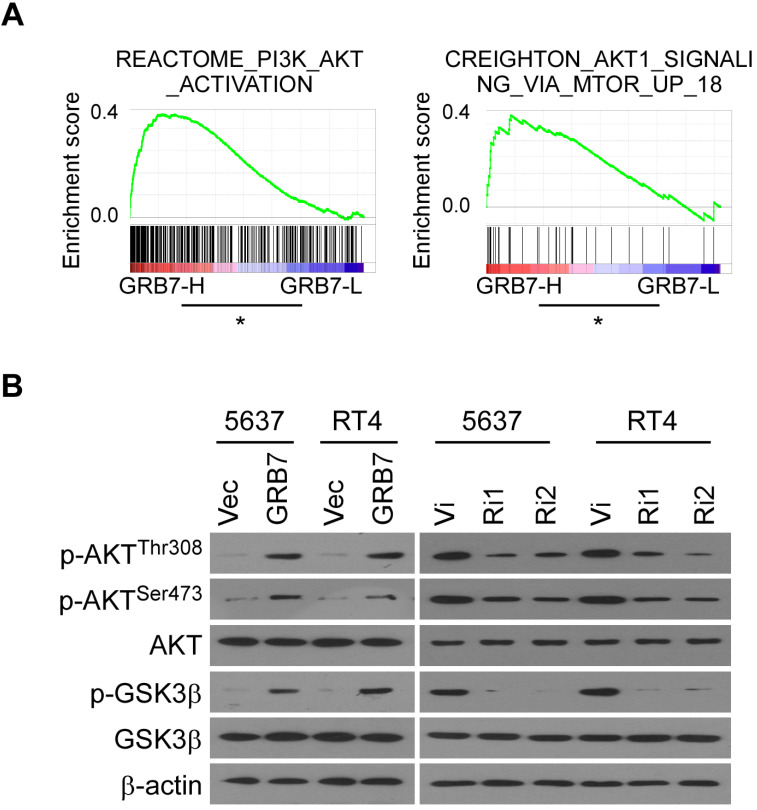
** Phospho-AKT pathway is related to GRB7 expression. A.** GSEA plot showing that the GRB7 RNA level positively correlated with AKT-activated gene signatures. **B.** Western blot analysis detected the AKT activation associated proteins the indicated bladder cancer cells. Three independent experiments were conducted. *P < 0.05.

GSEA analysis showed that RNA levels of GRB7 are positively correlated with AKT-activated gene signature (Figure 5A). For experimental validation, we first examined the phosphorylation status that positively determines the level of AKT protein activity. As shown in Figure 5B, the key phosphorylation sites of AKT protein, threonine 308 and serine 473, were both phosphorylated in GRB7 overexpressing cells but dephosphorylated in GRB7 silenced cells. At the same time, the phosphorylation level of GSK3β, a recognized target protein downstream of AKT, also changed accordingly (Figure 5B). Taken together, these results indicate that GRB7 regulates AKT pathway activity.

### Phospho-AKT pathway mediates GRB7-induced cell proliferation and tumorigenesis

**Figure 6:**
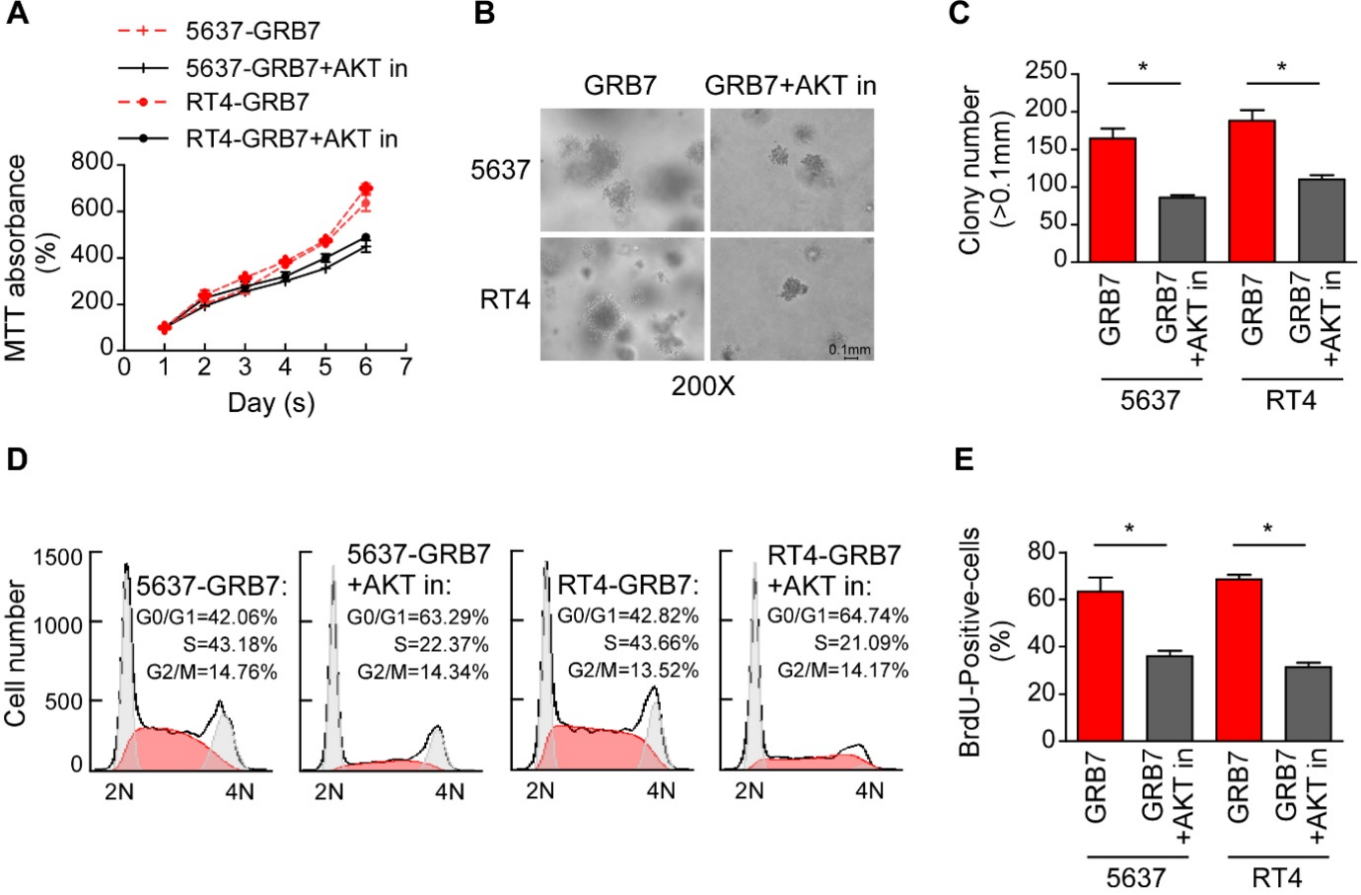
** Phospho-AKT pathway mediates GRB7-induced cell proliferation and tumorigenesis. A.** MTT assays showed that AKT inhibitor could significantly restore GRB7-promoted growth rate. **B.** The representative images of colonies in soft agar assays visually indicating that AKT inhibitors can weaken GRB7-induced tumorigenicity.** C.** Numbers of colonies in the anchorage-independent growth assay. **D.** Flow cytometry analysis showed that perifosine significantly abrogated the GRB7-induced enrichment of cells in the S phase.** E.** Quantification of BrdU incorporation in the constructed stable cell lines. *P < 0.05.

The effect of AKT on GRB7-induced cell proliferation was further verified by treatment of GRB7 overexpressing cells with the AKT inhibitor Perifosine. As shown in Figure 6A, the AKT inhibitor (AKT in) significantly slowed the growth of cells promoted by GRB7. Representative images and counts in the soft agar assay visually indicate that AKT inhibitors attenuate tumorigenesis induced by GRB7 (Figure 6B and 6C). Flow cytometry and BrdU labeling experiments showed that AKT inhibitors inhibited S-phase cell enrichment induced by GRB7, which also confirmed that AKT inhibitors can antagonize the regulation of GRB7 on cell cycle (Figure 6D and 6E). Taken together, phospho-AKT pathway mediates GRB7-induced cell proliferation and tumorigenesis in bladder cancer cells.

## Discussion

Detection of specific markers of abnormal expression in tissues is an important strategy for tumor diagnosis and treatment 20. As the earliest discovered tumor marker, alpha-fetoprotein (AFP) has become the main screening index for primary liver cancer 21. Urinary elevated human chorionic gonadotropin (HCG) in patients with malignant teratoma is associated with prognosis 22. A variety of drugs that target abnormal genes in tumors, such as vascular endothelial growth factor receptor inhibitors Bevacizumab, anti-HER2 monoclonal antibodies Trastuzumab, Bcr-abl tyrosine kinase inhibitors Imatinib and the like have been clinically applied and have achieved good therapeutic effects 23-25. Many studies have been devoted to the search for new molecular targets for bladder cancer. For example, high expression of sirtuin 1 significantly promoted the proliferation of bladder cancer cells 26. High expression of miRNA-373 promoted tumor cell migration and up-regulated EGFR expression 27. This study found that the expression of GRB7 is elevated in human bladder cancer, and the abnormal expression of GRB7 promotes the proliferation and tumorigenesis of bladder cancer cells by promoting cell cycle G1/S transition. This finding further consolidates the theoretical basis for GRB7 as a therapeutic target for a variety of tumors. The development of targeted GRB7 is already underway. An 11-residue thioether-cyclized peptide known as G7-18NATE has been developed which inhibits GRB7 by specific interaction with the SH2 domain 28. Using a virtual screening strategy, nine new benzamide-based GRB7-SH2 domain antagonists were identified and lead compound 1 was found to inhibit MDA-MB-468 breast cancer cell growth 29. Clearly, studying the structure of GRB7 and the proteins it interacts with are critical for the development of anticancer drugs that target GRB7.

Previous studies have found that GRB7 binds to PIP3 (Phosphatidyl Inositol-3-Phosphate) via its PH domain 30. It is well known that PIP3 binds to and promotes phosphorylation of the Ser308 site of the AKT protein, which is also a pre-requisite for AKT kinase activation 31. Activation of AKT kinase plays a very important role in the malignant progression of bladder cancer 32. It has been reported that regulation of AKT activity induces a cellular response to tumor necrosis factor-related apoptosis-inducing ligand (TRAIL) 33; AKT signaling is involved in the regulation of inflammation and tumor formation in bladder cancer; activation of AKT increases tumor cell resistance to paclitaxel 34. Given the importance of AKT in bladder cancer, we sought to investigate the role of GRB7 in AKT signaling. Our current study found that AKT phosphorylation levels are elevated in cells overexpressing GRB7, but are reduced in cells that are silenced by GRB7. Based on this, it is speculated that AKT signaling is involved in GRB7-induced cell proliferation and tumorigenesis. However, whether the activation of AKT protein by GRB7 protein is directly or indirectly is still to be further studied. In addition, why GRB7 is elevated in bladder cancer is also a problem worth studying. It has been reported that GRB7 is inhibited by the PI3K-AKT pathway, suggesting that there may be a negative feedback between GRB7 overexpression and AKT activation. Silencing of GRB7 increased the role of the AKT inhibitor Lapatinib in breast cancer, which also supports the role of GRB7 in AKT activity, and also suggests that targeting GRB7 is expected to be a combination of drugs targeting AKT 35.

In general, this study shows that GRB7 is upregulated in human bladder cancer and plays an important role in promoting proliferation and tumorigenesis via phosphorylated-AKT pathway, which suggests a potential role of GRB7 as a diagnostic marker and valuable therapeutic target in bladder cancer.

## Abbreviations

BC: Bladder Cancer
GRB7: Growth factor receptor-bound protein 7
TCGA: The Cancer Genome Atlas
RT-qPCR: Real-time Quantitative PCR
GSEA: Gene Set Enrichment Analysis
BTA: bladder tumor antigen
BLCA-4: nuclear matrix protein
CEA: carcinoembryonic antigen
CA-125: carbohydrate chain antigen 125
BrdU: Bromodeoxyuridine
NOD/SCID: Non-obese diabetic/severe combined immunodeficiency
MTT: 3-(4,5-Dimethyl-2-thia-zolyl)-2,5-diphenyl-2-H-tetrazolium bromide
DMSO: dimethyl sulfoxide
ErbB: Erythroblastic Leukemia Viral Oncogene Homolog

## References

[1] Ge P, Wang L, Lu M, Mao LJ, Li W, Wen RM (2018). Oncological Outcome of Primary and Secondary Muscle-Invasive Bladder Cancer: A Systematic Review and Meta-analysis. Sci Rep-Uk.

[2] Clinton T, Lotan Y (2017). Review of the Clinical Approaches to the Use of Urine-based Tumor Markers in Bladder Cancer. Rambam Maimonides Med J.

[3] Chou R, Gore JL, Buckley D, Fu R, Gustafson K, Griffin JC (2015). Urinary Biomarkers for Diagnosis of Bladder Cancer: A Systematic Review and Meta-analysis. Ann Intern Med.

[4] Núria M, Aurelia B, Nascimento CM, Francisco F, Manuel R, Diana P (2005). P53 as a prognostic marker for bladder cancer: a meta-analysis and review. Lancet Oncol.

[5] Critelli R, Fasanelli F, Oderda M, Polidoro S, Assumma MB, Viberti C (2016). Detection of multiple mutations in urinary exfoliated cells from male bladder cancer patients at diagnosis and during follow-up. Oncotarget.

[6] Priolo G, Gontero P, Martinasso G, Mengozzi G, Tizzani AJCCA (2001). Bladder tumor antigen assay as compared to voided urine cytology in the diagnosis of bladder cancer. Science Direct.

[7] Szymanska B, Dlugosz A (2017). The role of the BLCA-4 nuclear matrix protein in bladder cancer. Postepy Hig Med Dosw (Online).

[8] Lamarca A, Barriuso J (2012). Urine telomerase for diagnosis and surveillance of bladder cancer. Adv Urol.

[9] Oliveira-Ferrer L, Tilki D, Ziegeler G, Hauschild J, Loges S, Irmak S (2004). Dual role of carcinoembryonic antigen-related cell adhesion molecule 1 in angiogenesis and invasion of human urinary bladder cancer. Cancer Res.

[10] Wiwanitkit V (2010). CA125 for following up carcinoma of the bladder. Urol Oncol.

[11] Lucas-Fernandez E, Garcia-Palmero I, Villalobo A (2008). Genomic organization and control of the grb7 gene family. Curr Genomics.

[12] Shen TL, Guan JL (2004). Grb7 in intracellular signaling and its role in cell regulation. Front Biosci.

[13] Paudyal P, Shrestha S, Madanayake T, Shuster CB, Rohrschneider LR, Rowland A (2013). Grb7 and Filamin-a associate and are colocalized to cell membrane ruffles upon EGF stimulation. J Mol Recognit.

[14] Pero SC, Oligino L, Daly RJ, Soden AL, Liu C, Roller PP (2002). Identification of novel non-phosphorylated ligands, which bind selectively to the SH2 domain of Grb7. J Biol Chem.

[15] Mi JK, Kim RN, Song K, Jeon S, Jeong HM, Kim JS (2017). Genes co-amplified withERBB2orMETas novel potential cancer-promoting genes in gastric cancer. Oncotarget.

[16] Liu BY, Cao G, Dong Z, Chen W, Xu JK, Zhang SL (2016). [Knockdown of Grb7 inhibits growth of oral squamous cell carcinoma, cell proliferation and promoted apoptosis through ERK/FOXM1 pathway]. Shanghai Kou Qiang Yi Xue.

[17] Bivin WW, Yergiyev O, Bunker ML, Silverman JF, Krishnamurti U (2017). GRB7 Expression and Correlation With HER2 Amplification in Invasive Breast Carcinoma. Appl Immunohistochem Mol Morphol.

[18] Zhao HB, Zhang XF, Jia XL, Wang HB (2017). Grb7 is over-expressed in cervical cancer and facilitate invasion and inhibit apoptosis in cervical cancer cells. Pathol Res Pract.

[19] Song L, Wang L, Li Y, Xiong H, Wu J, Li J (2010). Sam68 up-regulation correlates with, and its down-regulation inhibits, proliferation and tumourigenicity of breast cancer cells. J Pathol.

[20] Sun YW, Li XH, Wang H, Wu J (2019). MiR-431 is a prognostic marker and suppresses cell growth, migration and invasion by targeting NOTCH2 in melanoma. Eur Rev Med Pharmacol Sci.

[21] Li Z, Wu Y, Zhou D (1996). [Antibody-targeted radio-chemotherapy of human primary liver cancer with radio-iodinated AFP monoclonal antibodies conjugated with mitomycin C: a preliminary clinical observation]. Zhonghua Zhong Liu Za Zhi.

[22] Fan J, Wang M, Wang C, Cao Y (2017). Advances in human chorionic gonadotropin detection technologies: a review. Bioanalysis.

[23] Kini SD, Yiu DW, Weisberg RA, Davila JF, Chelius DC (2019). Bevacizumab as Treatment for Epistaxis in Hereditary Hemorrhagic Telangiectasia: A Literature Review. Ann Otol Rhinol Laryngol.

[24] Wu S, Quan R, Han L (2019). Trastuzumab-based therapy is effective for salivary duct carcinoma: Case report and review of the literature. Oral Oncol.

[25] Navarrete-Dechent C, Mori S, Barker CA, Dickson MA, Nehal KS (2019). Imatinib Treatment for Locally Advanced or Metastatic Dermatofibrosarcoma Protuberans: A Systematic Review. JAMA Dermatol.

[26] Chen J, Cao L, Li Z, Li Y (2019). SIRT1 promotes GLUT1 expression and bladder cancer progression via regulation of glucose uptake. Hum Cell.

[27] Wang Y, Xu Z, Wang X (2019). miRNA-373 promotes urinary bladder cancer cell proliferation, migration and invasion through upregulating epidermal growth factor receptor. Exp Ther Med.

[28] Gunzburg MJ, Ambaye ND, Del Borgo MP, Perlmutter P, Wilce JA (2013). Design and testing of bicyclic inhibitors of Grb7-are two cycles better than one?. Biopolymers.

[29] Ambaye ND, Gunzburg MJ, Lim RC, Price JT, Wilce MC, Wilce JA (2013). The discovery of phenylbenzamide derivatives as Grb7-based antitumor agents. ChemMedChem.

[30] Shen TL, Han DC, Guan JL (2002). Association of Grb7 with phosphoinositides and its role in the regulation of cell migration. J Biol Chem.

[31] Xie Y, Shi X, Sheng K, Han G, Li W, Zhao Q (2019). PI3K/Akt signaling transduction pathway, erythropoiesis and glycolysis in hypoxia (Review). Mol Med Rep.

[32] Jiang L, Dong P, Zhang Z, Li C, Li Y, Liao Y (2015). Akt phosphorylates Prohibitin 1 to mediate its mitochondrial localization and promote proliferation of bladder cancer cells. Cell Death Dis.

[33] Shrader M, Pino MS, Lashinger L, Bar-Eli M, Adam L, Dinney CP (2007). Gefitinib reverses TRAIL resistance in human bladder cancer cell lines via inhibition of AKT-mediated X-linked inhibitor of apoptosis protein expression. Cancer Res.

[34] Li Y, Chen K, Li L, Li R, Zhang J, Ren W (2015). Overexpression of SOX2 is involved in paclitaxel resistance of ovarian cancer via the PI3K/Akt pathway. Tumour Biol.

[35] Nencioni A, Cea M, Garuti A, Passalacqua M, Raffaghello L, Soncini D (2010). Grb7 upregulation is a molecular adaptation to HER2 signaling inhibition due to removal of Akt-mediated gene repression. PLoS One.

